# Cortical laminar necrosis in an infant with influenza A virus infection

**DOI:** 10.1002/ccr3.2936

**Published:** 2020-06-01

**Authors:** Kazufumi Yaginuma, Masahiro Watanabe, Yuichi Suzuki, Kazuhide Suyama, Koichi Hashimoto, Mitsuaki Hosoya

**Affiliations:** ^1^ Department of Pediatrics Fukushima Medical University Fukushima Japan

**Keywords:** cortical laminar necrosis, infant, influenza A virus

## Abstract

Cortical laminar necrosis comprises ischemic neuronal changes and glial reaction. Despite fewer reports in the pediatric population, we encountered a case of cortical laminar necrosis with influenza virus A infection in an infant.

## Graphical abstract

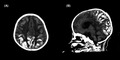

## 1 CASE PRESENTATION

Cortical laminar necrosis comprises ischemic neuronal changes and glial reaction. Although cortical laminar necrosis is a classic presentation related to conditions of energy depletion in adults, reports concerning children are few.

Herein, we report cortical laminar necrosis with influenza virus A infection in an infant.

Cortical laminar necrosis comprises ischemic neuronal changes and glial reaction. Ischemic neuronal changes involve the third layer of cerebral cortex, and laminar deposition of fat‐laden macrophages accompanies glial reaction.^1^, ^2^ Reports of cortical laminar necrosis in children are few.

**Figure 1:**
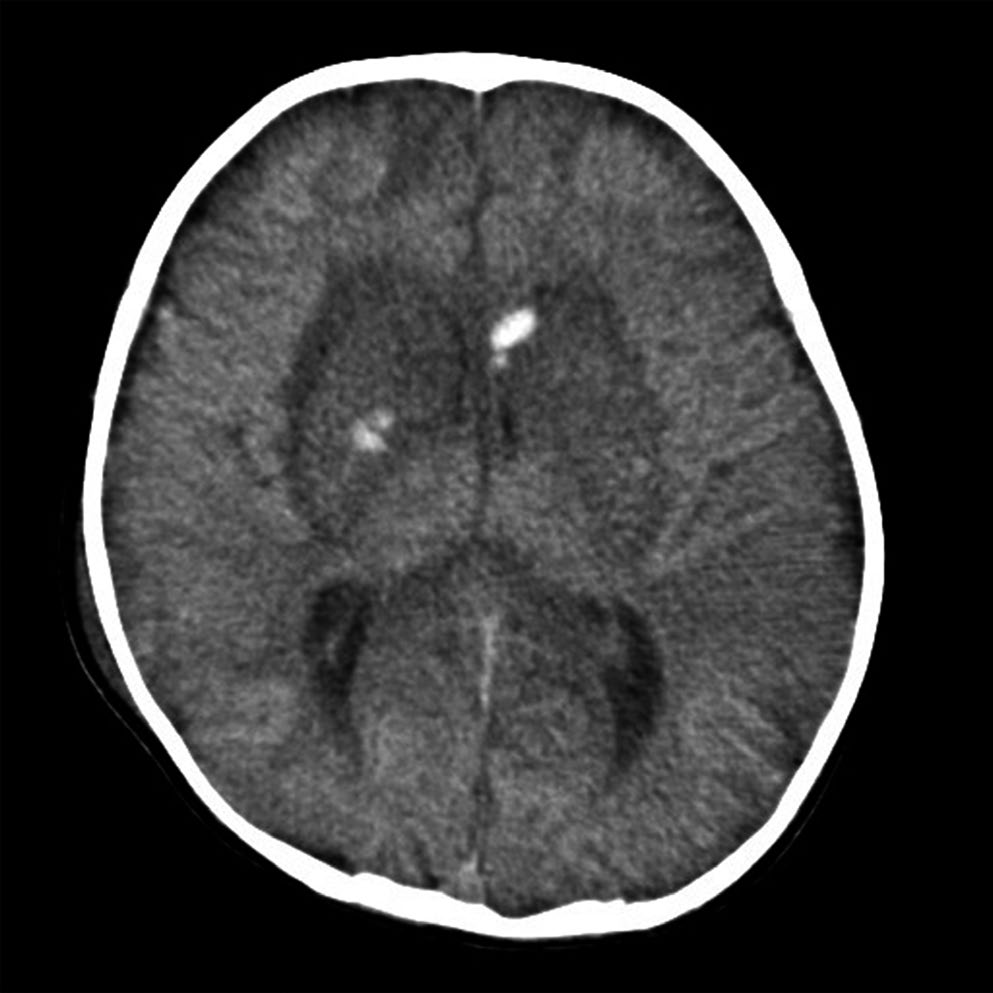
Computed tomography performed 2 wks after admission showing overall high‐density lesions in the cerebral cortex and hemorrhage in the bilateral basal ganglia (arrows)

**Figure 2:**
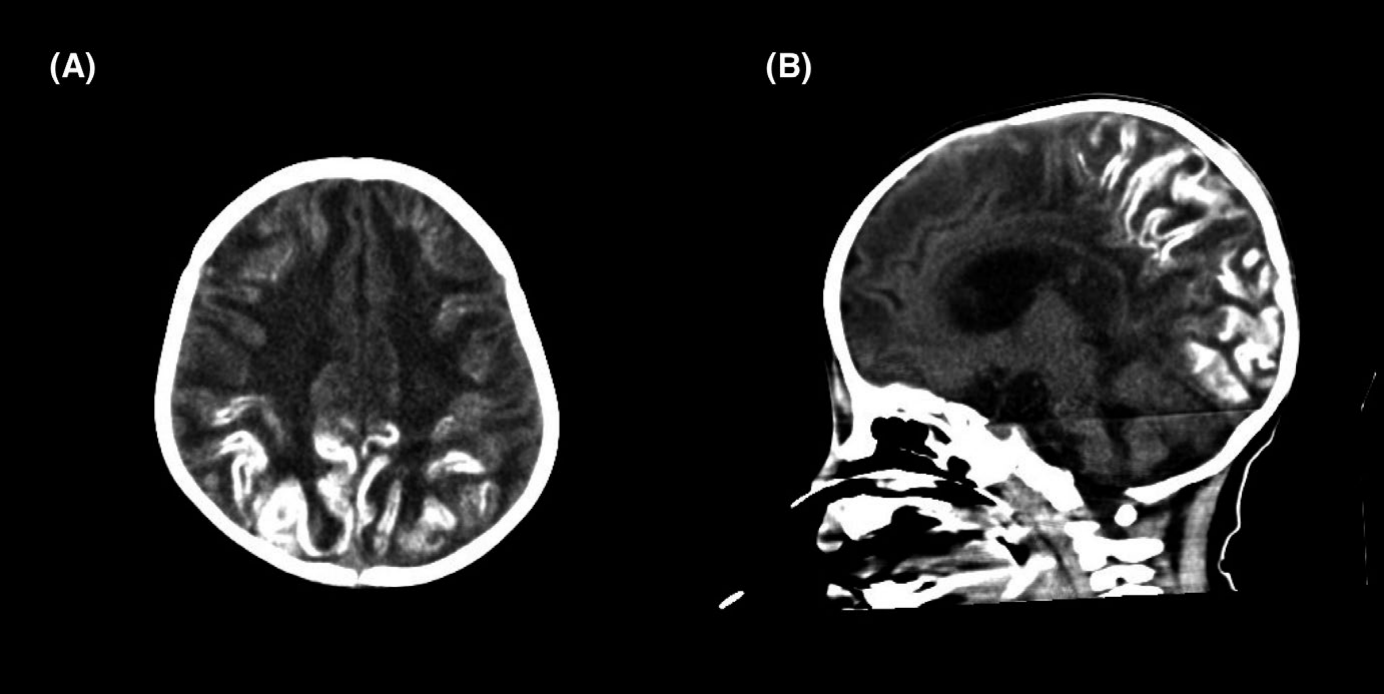
A, Axial computed tomography performed 1 mo after admission showing high‐density lesions in the temporoparietal regions. B, Sagittal computed tomography showing high‐density lesions in the parieto‐occipital regions

A 1‐year‐old boy with a medical history of ventricular septum defect developed status epilepticus and shock. Blood chemistry at admission revealed aspartate transaminase, 239 U/L; alanine transaminase, 57 U/L; lactate dehydrogenase, 1063 U/L; and ferritin 3381 ng/mL. These results indicated that the patient may have hypercytokinemia. Real‐time polymerase chain reaction using nasopharyngeal swab samples was positive for the influenza A virus; he was clinically diagnosed with influenza virus–associated acute encephalitis/encephalopathy. Oseltamivir phosphate and methylprednisolone pulse therapy was administered. Head computed tomography (CT) at admission showed loss of gray‐white differentiation. Follow‐up CT performed 2 weeks after admission revealed overall hyperdensity in the cerebral cortex and hemorrhage in the bilateral basal ganglia (Figure 1). Subsequent follow‐up CT performed a month later showed laminar hyperdensity around the cortical gyri, mostly in the temporo‐parieto‐occipital region (Figure 2).

These results enabled the diagnosis of cortical laminar necrosis with bilateral deep gray nuclei involvement as a manifestation of influenza virus–associated acute encephalitis/encephalopathy and anoxic‐ischemic brain injury.

## CONFLICT OF INTEREST

The authors declare that there is no conflict of interest regarding the publication of this article.

## AUTHOR CONTRIBUTIONS

KY: made substantial contributions to the study's conception and design, and acquisition, analysis, and interpretation of data. MW, YS, KS, KH, and MH: were involved in drafting or revising the manuscript critically for important intellectual content.
